# Accuracy of robotic patient positioners used in ion beam therapy

**DOI:** 10.1186/1748-717X-8-124

**Published:** 2013-05-21

**Authors:** Olaf Nairz, Marcus Winter, Peter Heeg, Oliver Jäkel

**Affiliations:** 1 Heidelberger Ionenstrahl-Therapiezentrum, Im Neuenheimer Feld 450, Heidelberg 69120, Germany; 2 Department of Radiation Oncology and Radiotherapy, University Hospital Heidelberg, Im Neuenheimer Feld 400, Heidelberg 69120, Germany; 3 Paracelsus Medical University Salzburg, Strubergasse 21, Salzburg 5020, Austria

**Keywords:** Robotic patient positioning, 6 degrees of freedom, Accuracy, Ion beam therapy, High precision radiation therapy

## Abstract

**Background:**

In this study we investigate the accuracy of industrial six axes robots employed for patient positioning at the Heidelberg Ion Beam Therapy Center.

**Methods:**

In total 1018 patient setups were monitored with a laser tracker and subsequently analyzed. The measurements were performed in the two rooms with a fixed horizontal beam line. Both, the 3d translational errors and the rotational errors around the three table axes were determined.

**Results:**

For the first room the 3d error was smaller than 0.72 mm in 95 percent of all setups. The standard deviation of the rotational errors was at most 0.026° for all axes. For the second room Siemens implemented an improved approach strategy to the final couch positions. The 95 percent quantile of the 3d error could in this room be reduced to 0.53 mm; the standard deviation of the rotational errors was also at most 0.026°.

**Conclusions:**

Robots are very flexible tools for patient positioning in six degrees of freedom. This study proved that the robots are able to achieve clinically acceptable accuracy in real patient setups, too.

## Background

With the increasing dose conformity achieved in modern radiation therapy techniques the requirements on the accuracy of patient positioning are becoming tighter as well. This is especially true for ion beam therapy where in general the dose gradients are steeper and the dose deposition has a stronger dependency on the materials traversed than in conventional radiation therapy with photons ( [1-4]). Therefore patient setup for therapy should be an exact replication of the setup for the planning CT, which makes an accurate setup correction in all six degrees of freedom necessary ( [5,6]). Hence especially at particle therapy centers the use of robots for patient positioning attracted attention already many years ago ( [7-9]). First prototypes were developed and several patents issued (e.g. [10]).

Robots are known for their high reproducibility, i.e. they are able to reach the same position with high precision under the same load. In this study we investigate whether the industrial six axes robots used at the Heidelberg Ion Beam Therapy Center (HIT) are also able to execute the movements precisely with patients having different weights and target points in different locations of the treatment volume. The robots’ movements where monitored during therapy in real patient setups with an external laser tracker. The results presented here can therefore serve in clinical practice as an input for calculating the required safety margins to the clinical target volumes.

## Methods

### The patient positioner

**Figure 1:**
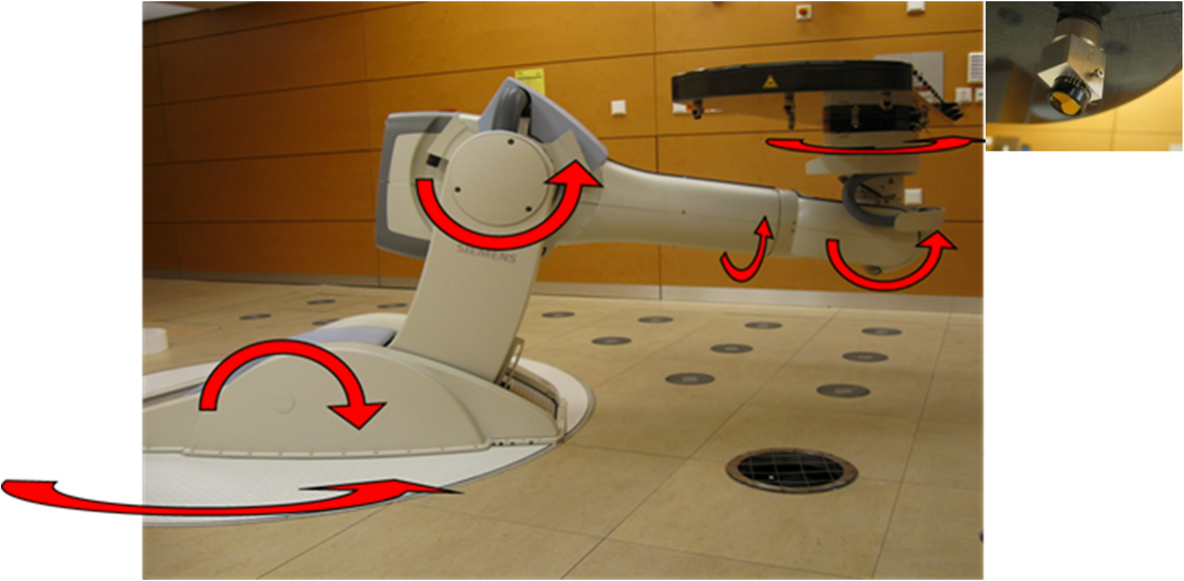
**Six axes robots employed at the Heidelberg Ion beam therapy centre for patient positioning.** The laser tracker for monitoring the movements of the couch is located under the false floor beneath the steel grid. The retroreflectors can be seen at the bottom side of the couch (see also small picture on the right).

**Figure 2:**
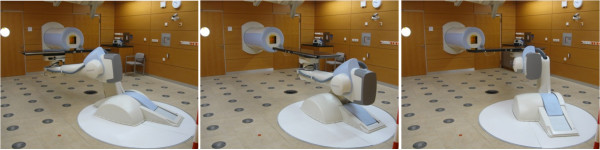
**Treatment table at 180° (left), 270° (middle) and 0° (right).** In most of the cases presented here the imaging for position verification was performed at 270°.

For the ion beam therapy center in Heidelberg Siemens Medical Solutions adapted floor mounted KUKA robots (KR 240 L210 MED from KUKA, Augsburg, Germany) to carry the treatment couch (Figures 1 and 2). The two robots investigated are installed in the treatment rooms with fixed horizontal beam lines. They allow data entry and therefore minimum movements of 0.1 millimeters and 0.1 degrees.

Rotations around the table’s longitudinal axis (roll) and lateral axis (pitch) up to 15° are in principle possible but are confined in the present version to 5°. The range of possible isocentric angles, i.e. angles resulting from rotations around the vertical axis, amounts to 200°. The patient table is in line with the beam axis at an angle of 270°; from there isocentric rotations of +/- 100° (to 170° and 10°) are possible.

**Figure 3:**
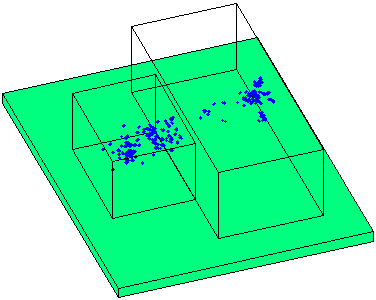
**Treatment volume and target points.** The target volume consisting of a cranial (smaller cuboid) and a caudal part and all target points of this study (in blue) are shown. In green a part of the treatment table top is sketched.

The treatment volume, which is the sum of all possible target points, is composed of two cuboids (Figure 3). The cranial part, which comprises the first 26 cm, measures 21 cm in width and 30 cm in height. The caudal part has a width of 45 cm and a height of 35 cm. In total the treatment volume has a length of 59 cm.

### The measuring device

In order to monitor the movements of the couch we used a laser tracker – an optical measuring device, which is able to measure the position of a retroreflector (target) with a precision of typically a few tens of micrometers ( [11]). The laser tracker was mounted under the false floor beneath the isocenter, the targets were located on the bottom side of the couch (Figure 1). A hole in the tiles of the floor was kept open above the laser tracker. We chose the diameter of the hole and the mounting angle of the targets in a way that each target could be seen by the laser tracker for all possible treatment positions of the couch. In total we used six retroreflectors. All of them were located beyond the imaging area, three of them caudally and three of them cranially, so that they did not interfere with X-ray images acquired for position verification.

During the treatment sessions a coarse grid of thin but robust steel wires covered the hole in the floor to protect personnel from tripping into it. This grid, however, hides some of the targets for certain table positions. As long as in total at least three targets and at least one on the caudal and one on the cranial side were detected by the tracker, the position of the couch could still be determined.

### Workflow at HIT

At the time of the treatment planning CT, skin marks or marks on the patient’s mask are drawn to indicate the position of the reference point. This point is in general not equivalent to the target point, which is determined in the treatment planning system and is typically set in the middle of the target volume. For our (isocentric) treatment technique the target point has to be at the isocenter for irradiation.

At the first treatment session the patient marks are aligned with the room lasers. The table coordinates of this position are stored for all future sessions. Then the known offset to the target point is applied.

Position verification is performed with a C-arm X-ray imaging system (Axiom Artis from Siemens, Germany), which is carried by a ceiling mounted robot. For imaging the robot brings the C-arm down from the parking position at the ceiling to the imaging position at isocenter. The X-ray robot can perform full 360° rotation around the table’s longitudinal axis at isocenter height for almost all table angles. The patient positioning is assessed by two, in general orthogonal, images. In most cases this is done at an isocentric table angle of 270° (Figure 2), i.e. when the longitudinal axis of the table is parallel to the beam axis.

For determining the setup correction vector an automated 2D-3D matching algorithm is employed. Therefore DRRs (digitally reconstructed radiographs) calculated from the treatment planning CT are matched to the planar images. The algorithm finds that transformation (rotational and translational) of the treatment planning CT that minimizes the difference between the calculated DRRs and the X-ray images. The match between the DRRs and the X-ray images is visually checked by the radiation therapists and possible errors are eliminated by manually applied corrections to the registration. The so determined setup correction in all six degrees of freedom is applied together with the isocentric table rotation to the first treatment position.

For the patients in this study the required dose was applied by one to three fields at different table angles. Patients treated for head tumors, all of them immobilized with a mask [12], were typically imaged only at the beginning of the treatment workflow at 270°. Pelvic patients were imaged before the delivery of each beam, in general in the planned treatment position. The location of all target points in this study is shown in Figure 3.

### Measurements

The measurements were performed with a FARO laser tracker, model X (FARO Technologies Inc, USA). FARO provided a software development kit which enabled us to write our own program for controlling and reading out the tracker during patient treatment. A graphical user interface (GUI) implemented in MATLAB allowed the input of all necessary data like table coordinates and patient names.

Assuming that the relation between target and table coordinates has been established for one table position, the nominal positions of the targets can be calculated for all other table coordinates. The nominal values serve as a starting point for the search of the actual positions of the targets. Differences between the actual and nominal coordinates can be of the order of one millimeter, depending mainly on the patient load.

The measurements can be started from the GUI. As soon as the tracker finds a target, the target coordinates are recorded and the search for the next target starts. In case that the tracker cannot find a target – e.g. because it is hidden behind one of the wires of the grid – the tracker aborts the search for this target and continues with the next. The search for all six targets takes typically 30 seconds, which is far less than the irradiation time of one field.

The radiation therapists were asked to perform a measurement in both the verification position, i.e. the position in which the imaging was performed, and in each treatment position. Since no data exchange between the patient record and verify system and the software for the laser tracker measurements is possible, patient names and table coordinates had to be typed in by the therapists manually. The table coordinates are needed to calculate the nominal positions of the targets and hence the starting point of the laser tracker’s search, the patient names for assigning the measurements accordingly. When a measurement is completed the software records furthermore the measured target coordinates and the measurement time. All manually typed coordinates were in a later step compared to the values from the treatment records as stored by the patient record and verify system and corrected if necessary. The correction of the manual inputs is important to exclude that possible typos could fake inaccuracies of the robots.

### Calculating the performed table movements

In order to determine the performed table movements both the measured coordinates of the targets in the verification and in the treatment position are required. The actually performed table movements can be calculated by finding the transformation between the measured target coordinates for the two table positions. This transformation consists of rotations and translations in the table coordinate system. The laser tracker, however, measures the target positions in the fixed room coordinate system (IEC fixed). So also the sought transformation between the target coordinates have to be calculated in the fixed system.

In general a rotation by an angle α_axis_ about an axis in the direction of a unit vector

$$n_{\mathrm{axis}}=\left(\begin{array}{c} n_{1}\\ n_{2}\\ n_{3} \end{array}\right)$$

is given by the rotation matrix

$$R_{n_{\mathrm{axis}},\alpha_{\mathrm{axis}}}=\left(\begin{array}{ccc} \cos\left(\alpha \right)+n_{1}^{2}\left(1-\cos\left(\alpha \right)\right) & n_{1}n_{2}\left(1-\cos\left(\alpha \right)\right)-n_{3}\sin\left(\alpha \right) & n_{1}n_{3}\left(1-\cos\left(\alpha \right)\right)+n_{2}\sin\left(\alpha \right)\\ n_{1}n_{2}\left(1-\cos\left(\alpha \right)\right)+n_{3}\sin\left(\alpha \right) & \cos\left(\alpha \right)+n_{2}^{2}\left(1-\cos\left(\alpha \right)\right) & n_{2}n_{3}\left(1-\cos\left(\alpha \right)\right)-n_{1}\sin\left(\alpha \right)\\ n_{1}n_{3}\left(1-\cos\left(\alpha \right)\right)-n_{2}\sin\left(\alpha \right) & n_{2}n_{3}\left(1-\cos\left(\alpha \right)\right)+n_{1}\sin\left(\alpha \right) & \cos\left(\alpha \right)+n_{3}^{2}\left(1-\cos\left(\alpha \right)\right) \end{array}\right)$$

For the isocentric rotation by an angle α_iso_ the unit vector is in our coordinate system convention

$$n_{\mathrm{iso}}=\left(\begin{array}{c} 0\\ 0\\ 1 \end{array}\right)$$

The rotation axis of the pitch for the isocentrically rotated system is given by

$$n_{\mathrm{pitch}}=R_{n_{\mathrm{iso}},\alpha_{\mathrm{iso}}}R_{n_{\mathrm{iso}},\alpha_{\mathrm{PV}}}\left(\begin{array}{c} 1\\ 0\\ 0 \end{array}\right)$$

where α_PV_ is the isocentric angle where the position verification images were taken and α_iso_ the rotation angle between position verification and treatment position.

Finally the unit vector for roll is given by

$$n_{\mathrm{roll}}=R_{n_{\mathrm{pitch}},\alpha_{\mathrm{pitch}}}R_{n_{\mathrm{iso}},\alpha_{\mathrm{iso}}}R_{n_{\mathrm{iso}},\alpha_{\mathrm{PV}}}\left(\begin{array}{c} 0\\ 1\\ 0 \end{array}\right)$$

Hence the transformation between the target coordinates in the position verification position T_PV_ and the setup corrected treatment position T_tr_ is given by

$$T_{\mathrm{tr}}=R_{n_{\mathrm{roll}},\alpha_{\mathrm{roll}}}R_{n_{\mathrm{pitch}},\alpha_{\mathrm{pitch}}}R_{n_{\mathrm{iso}},\alpha_{\mathrm{iso}}}R_{n_{\mathrm{iso}},\alpha_{\mathrm{PV}}}T_{\mathrm{PV}}+v$$

with v being the translational setup correction vector in the room coordinate system.

The six parameters defining the transformation (α_iso,_ α_pitch,_ α_roll_ and the three components of the vector v) are found by fitting the measured target coordinates of the treatment position to the rotated and translated target coordinates of the verification position calculated with the formula above. The result of the fitting gives the executed movement of the robot. In a final step the executed table movements are compared with the intended table movements and the error in each of the six components is determined.

## Results and discussion

In order to assess the accuracy of the robots we analyzed in total 1018 setups, 512 in the first horizontal treatment room (H1) performed from June to September 2010 and 506 in the second horizontal treatment room (H2) from October 2010 to December 2010. The robot hardware is the same for both rooms but for H2 Siemens implemented a different approach strategy to the target position, which avoided inaccuracies due to the backlash of the axes. The cumulated effect of the backlash for all six axes on the target position may amount to several hundred microns. In order to eliminate this effect, each robot movement to any target position is followed by an incremental, axis specific back and forth movement, which ensures that all axis specific movements are always in the same direction at the end of the move. The incremental movement has to be greater than the backlash of the axes. In our case the order of magnitude is well below 1° and the resulting movement of the table top is hardly recognizable by the patient.

**Figure 4:**
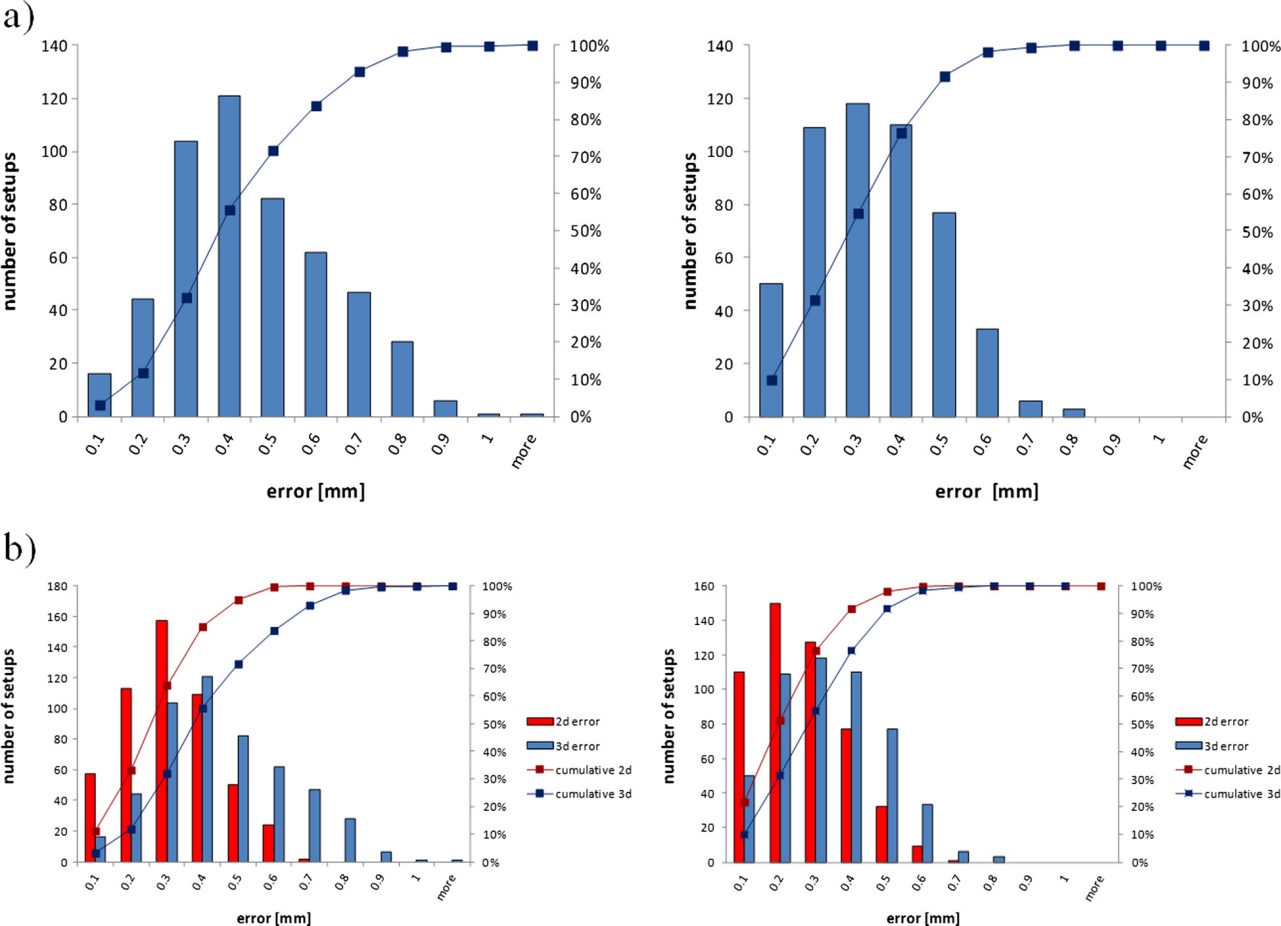
**a**: **Histograms of the measured 3d error in patient setups in room H1 (left) and H2 (right) .** The solid lines show the cumulative error. **b**: For comparison the measured 2d error (orthogonal to the beam) are shown in red. Left for room H1, right for H2.

Figure 4a shows the histograms of the measured errors in 3d for both rooms, all the numerical results of the measurements are summarized in Table 1. As can be seen from the data the implemented approach strategy in H2 proved to be effective. The median 3d error was 0.38 mm for H1 and 0.28 mm for H2, the 95 percent quantile 0.72 mm for H1 and 0.53 mm for H2, i.e. in H2 in 95 percent of all setups the accuracy of the robot’s movements was equal or better than 0.53 mm.

**Table 1:** Summary of the measurement results

		**Room H1**	**Room H2**
Number of setups		512	506
3d error	median	0.38 mm	0.28 mm
	**0.95 quantile**	**0**.**72 mm**	**0**.**53 mm**
	maximum	1.02 mm	0.75 mm
2d error	median	0.25 mm	0.20 mm
	**0.95 quantile**	**0**.**50 mm**	**0**.**43 mm**
	maximum	0.66 mm	0.62 mm
rotational error	isocentric mean	−0.007°	−0.006°
	*isocentric standard deviation*	*0*.*026*°	*0*.*019*°
	pitch mean	−0.010°	−0.022°
	*pitch standard deviation*	*0*.*020*°	*0*.*014*°
	roll mean	−0.015°	−0.012°
	*roll standard deviation*	*0*.*025*°	*0*.*026*°

Since even larger setup errors along the beam direction hardly alter the dose distribution, the error orthogonal to the beam direction is of more importance than the 3d error. This error, we call it the 2d error, has its median value for H1 at 0.25 mm and 0.20 mm for H2. The 95 percent quantile amounts to 0.50 mm and 0.43 mm for H1 and H2, respectively. In Figure 4b both histograms, for the 2d and the 3d error, are shown for comparison.

**Figure 5:**
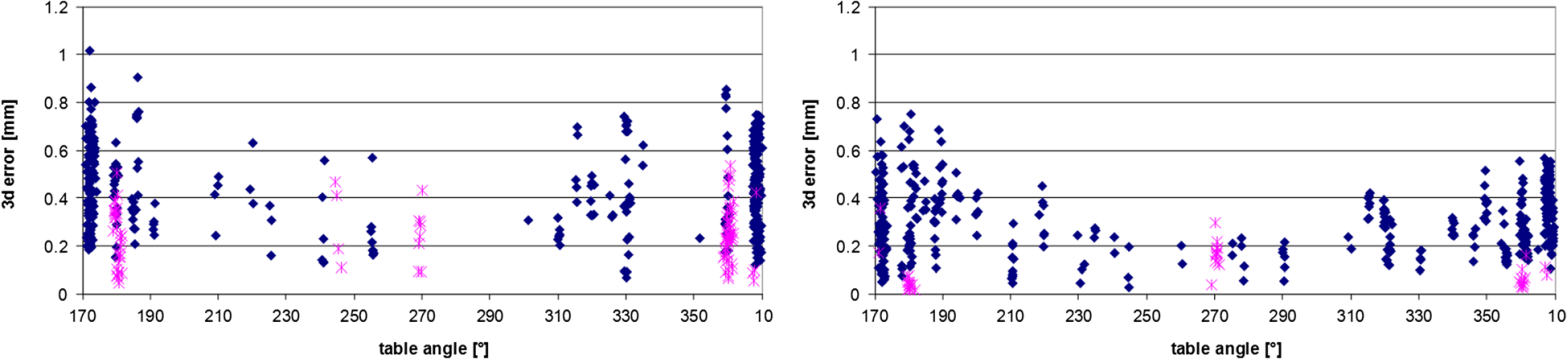
**Measured 3d error as a function of the table angle.** On the left the data from room H1, on the right from room H2 are shown. The setups where the verifications were done in the planned treatment positions are marked in pink. For the blue points the imaging was performed at a table angle of 270°.

It is also instructive to display the error as a function of the isocentric treatment angle (Figure 5). For most setups the imaging was not done in the planned treatment position but at a table angle of 270° (H1: 419 out of 512, H2: 475 out of 506). These positionings are marked in Figure 5 in blue; the others are shown in pink. The setups tend to be more accurate the smaller the isocentric rotations to the treatment positions are.

**Figure 6:**
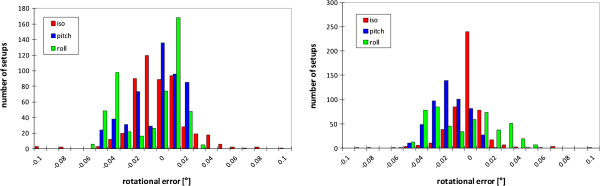
**Histogram of the measured rotational errors in patient setups in room H1 (left) and H2 (right).** Errors in rotations around the vertical axis are shown in red, around the left-right axis in blue and around the longitudinal axis of the table in green.

The histograms for the rotational errors around the three table axis are shown in Figure 6. The rotational errors turned out to be very small. The standard deviations of the measured errors are between 0.014° and 0.026°.

## Conclusions

Robots like the ones installed at HIT are very flexible tools for patient positioning in modern radiation therapy. They allow setup corrections in six degrees of freedom with a resolution of 0.1 mm and 0.1°. In this study we showed that the robots in spite of their high complexity perform their movements also with high precision. This was especially true after the new approach strategy to the final setup position was implemented.

In general the setups were performed more accurately when the verification was performed at a table angle close to the treatment angle. But since the accuracy of the analysed movements is comparable to the accuracy with which the X-ray position verification device can be calibrated, setup verification in treatment position does in general not increase the setup accuracy.

One has to admit that the initial calibration of the robots is a lengthy procedure. Due to different deflections under different loads the actual positions deviate from the nominal positions slightly. Therefore each robot has to be calibrated by first measuring this deviation for a subset of representative positions (in the order of some hundreds) and different loads ranging from 20 kg to 200 kg. In order to determine the correction for a specific load and position a model is applied, which inter- and extrapolates the deviations between the measured positions and loads.

In principle this calibration procedure can be avoided if an external tracking system is used not only for monitoring but also for controlling the movements of the robots. In this study it could be demonstrated that a commercial laser tracker would be a suitable tool to control patient setup in high precision radiation therapy. On the other hand it could also be shown that with a well calibrated system clinically acceptable accuracy can be achieved, too.

## Competing interests

The authors declare that they have no competing interests.

## Authors' contributions

All authors contributed to the design of the measurements and discussed the results. ON, MW and PH performed the setup. ON and MW performed the data analysis. ON drafted the manuscript. All authors read and approved the manuscript.
